# Condylar distances in hypermobile temporomandibular joints of patients with excessive mouth openings by using computed tomography

**DOI:** 10.4317/jced.51562

**Published:** 2014-12-01

**Authors:** Abbas Haghigaht, Amin Davoudi, Oleg Rybalov, Amin Hatami

**Affiliations:** 1 Assistant professor of Oral and Maxillofacial Surgery Dental Implants Research Center. Department of Oral and Maxillofacial Surgery, School of Dentistry, Isfahan University of Medical Sciences, Isfahan, Iran; 2 Dentistry student. Dental Students Research Center, School of Dentistry, Isfahan University of Medical Sciences, Isfahan, Iran; 3 MD professor of Oral and Maxillofacial Surgery. Department of Surgical Dentistry and Maxillofacial Surgery in plastic and reconstructive surgery of head and neck, Poltava, Ukraine; 4 Graduated Dentistry student. Torabinezhad Dental Research Center, School of Dentistry, Isfahan University of Medical Sciences, Isfahan, Iran

## Abstract

Objectives: hypermobility in Temporomandibular joint (TMJ) can manifest higher range of motions in mandible. The aim of this study was to compare the position and distances of the head of condyle to glenoid fossa in TMJs of healthy individuals and patients with mild, moderate and severe TMJ hypermobility. Material and Methods: In this clinical study, 69 patients (between the ages of 22 to 42) with manifestation of joint hypermobility were included and Computed tomography were administered for both TMJs. The patients were divided into three groups based on their maximum mouth opening (MMO): (A) with MMO of 50-55 mm; (B) with MMO between 55 to 65 mm; and (C) with MMO >65 mm. Also, 15 healthy people with profiled tomography in the last 6 months were assumed as control group (N) with normal MMO (<50 mm). The position of condyle from articular eminence while MMO; and the distances from anterior, superior and posterior border of condyle and facing wall of glenoid fossa were measured in closed mouth from the tomography of all contributors. The collected data were analyzed by one-way ANOVA, Post Hoc and Chi-Square tests using SPSS software version 15 at significant level of 0.05. Results: The superior and posterior distances were significantly higher in groups A, B and C than healthy individuals (all P values<0.01). The anterior distance was significant between groups B and N only in right TMJ (P=0.013). Conclusions: TMJ hypermobility showed the characteristic of increased condylar distance in posterior and superior specially in higher excessive mouth opening.

** Key words:**Computed tomography, joint hypermobility, mandibular condyle, mouth opening.

## Introduction

Internal deviations of Tempromandibular joint [TMJ] are discussed as a situation in which interferes with smooth movements of the mandible. Disc Displacements and hypermobility are suggested as the most common internal derangement of TMJ (1). Joint’s range of motion might be affected by numerous factors including: biochemical changes in the structure of collagen and elastin, loss of resistance to traction, laxity, increased joint mobility and generalized joint hypermobility [GJH] [a hereditary problem defined by an increase in range of motion in multiple joints (2). Also, the position of the head and body of mandible and emotional tensions may affect the bodily adaptations and realignments of tooth and TMJ (3,4).

Winocur E *et al.* conducted a study about prevalence of general joints laxity and TMJ hypermobility among adolescent girls. They concluded that the prevalence of generalized joints laxity was 43% and hypermobile TMJ was recognized in 27.3% (2).

In another survey, Adair SM *et al.* discovered that participants with GJH might be more likely to manifest some signs and symptoms of tempromandibular disorders than ones with normal mobility of joint (5). Also, Oral K *et al.* found that both local and general joints hypermobility are more diagnosed in patients with tempromandibular disorders, and the risk of TMJ dysfunction would be greater if the patients had both disorders simultaneously (6).

Computed tomography [CT] provides images of the bone components of TMJ with the advantages of demonstrating three-dimensional details (7). So due to the fact that TMJ hypermobility are reported as risk factors for tempromandibular disorders (5,6,8), the aim of present study was to compare the position and distances of head of condyle to glenoid fossa in TMJs of healthy individuals and patients with mild, moderate and severe TMJ hypermobility.

## Material and Methods

- Ethics: Present article is based on thesis with ID number of UDK:616.724-08.089.23; the survey was executed in medical and surgical department of Poltava Dental Clinic and Maxillofacial department of POKB, Ukraine; also a medical consent was filled by each contributors and all done procedures were required for treatment plans.

In this observational/case-control clinical study, 69 patients [between the ages of 22 to 42] with manifestation of TMJ hypermobility were included. Medical history and chief complaints were recorded from each patients and linear Computed tomography [Orthophos XG5, New York, USA] were administered for medial section of both left and right TMJs while maximum mouth opening [MMO] and closed mouth.

The exclusion criteria were: suffering from severe systematic diseases like rheumatoid arteritis and non-cooperative patients.

The patients were divided into three groups based on their MMO which was measured by positioning fingers between upper and lower incisors (9):

Group A: 25 patients with MMO of 50-55 mm.

Group B: 18 patients with MMO between 55 to 65 mm.

Group C: 26 patients with MMO >65 mm.

Also, we searched the tomography data base of oral and maxillofacial radiology department and we recalled 15 people with healthy TMJ who had taken tomography in the last 6 months for another reasons. The mentioned people assumed as control group [N] with recorded normal MMO [<50 mm] (9).

**Figure 1:**
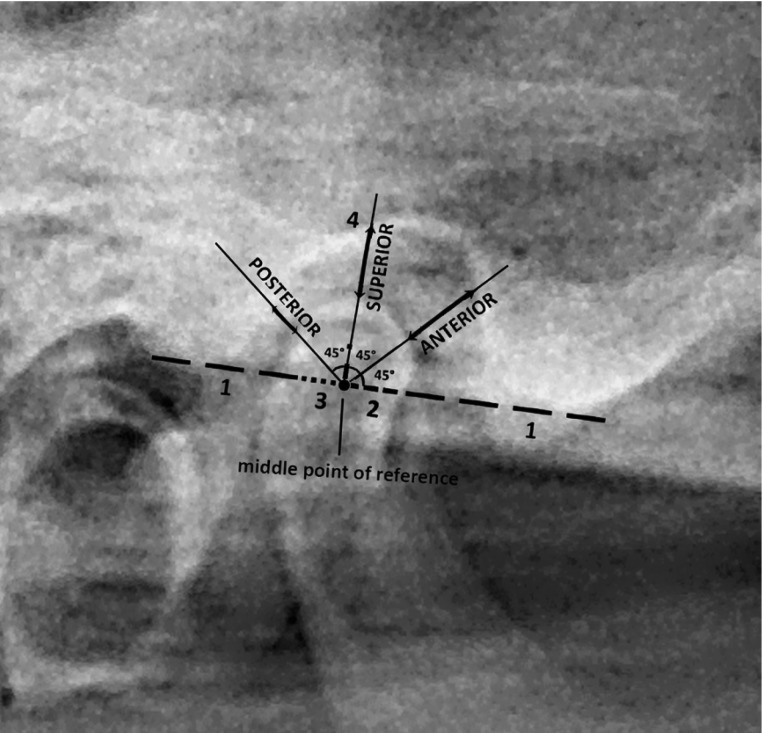
A tomography image from a hypermobile TMJ and way of measuring condylar distances.

In the next step, the magnification of 1.15% was considered and the lowest posterior and anterior extremities of the Tempromandibular fossa were assumed as reference line. An angle tool was used to form a 90° angle, which was then changed to a 45° angle from the reference line. Then the distance [mm] from anterior border of condyle head and facing wall of glenoid fossa was measured from the tomography (Fig. 1) by using Photoshop software CS6. This procedure was done for the posterior and superior border of condyle head for both TMJ of all groups during MMO; also the position of condyle from articular eminence was observed in closed mouth and reported as “behind”, “front” and “along”.

The collected data were analyzed by one-way ANOVA, Post Hoc and Chi-Square tests using SPSS software version 15 at significant level of 0.05.

## Results

The largest number of patients with TMJ hypermobility were at age of 31 to 42 years old [70.99%], while 37.67% were at the age of 26 to 35 years old. Also, the number of women was three times more [78.2%] than men [21.8%].

 Table 1 represents the descriptive results and  Table 2 reflects the comparisons with *P* values among different distances and groups which are highlighted as follow:

**Table 1:**
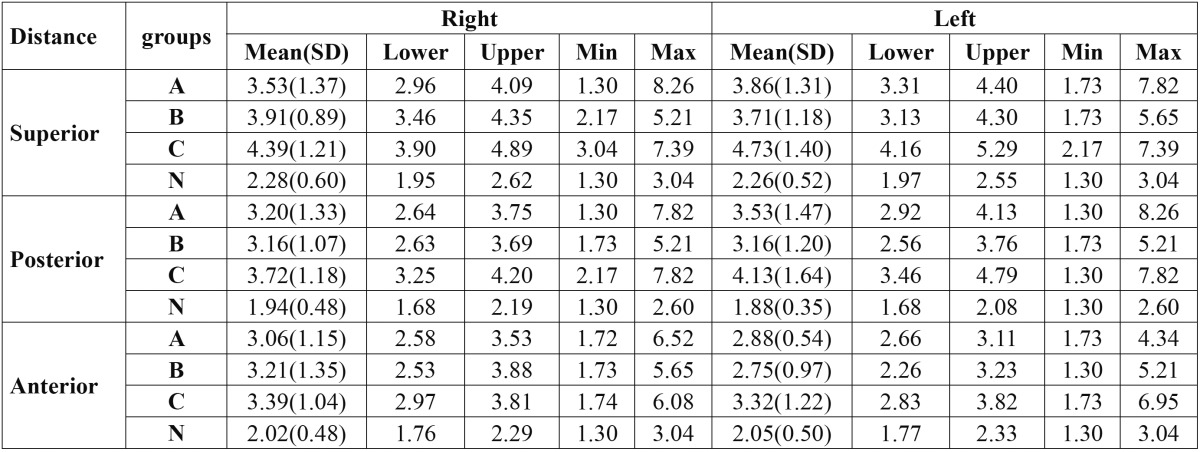
The descriptive analysis of measured anterior, superior and superior condylar distances (mm) of all the groups.

**Table 2:**
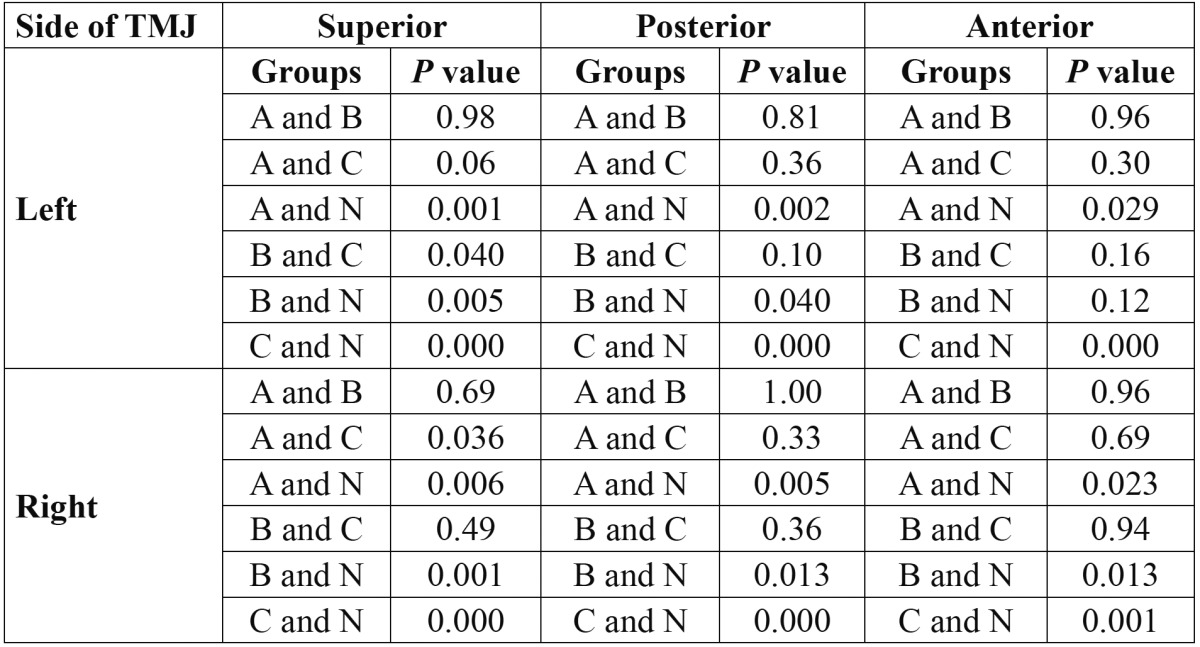
The comparisons and *P* values of measured anterior, superior and superior condylar distances among all the groups.

Superior distance:

Based on the results, the measured distances in groups A, B and C were significantly higher than group N in both TMJs [all *P* values<0.001]. But, the results showed significant differences between groups A and C only in the right TMJ [*P*=0.03]. Also, the distance between group B and C was significant only in the left TMJ [*P*=0.04].

Posterior distance:

The analyzed data revealed that the measured distances of groups A, B and C were in significant differences with group N in the both TMJs [all *P* values<0.01].

Anterior distance:

As the results represents, the mean distances in groups A and C were significantly higher than group N in the both TMJs [all *P* values<0.05]. But, the difference between group B and N was significant only in right TMJ [*P*=0.013].

Condyle position:

Based on the result showed in  Table 3, the condyle position was behind the articular eminence during MMO in large numbers of individuals in groups A and N in both TMJs. The both condyles were positioned along to the articular eminence in the highest proportion of patients of group B; and the position of the both condyles were mostly located in front of articular eminence in group C. Also, Pearson Chi-Square showed significant differences among groups [*P* value<0.001].

**Table 3:**
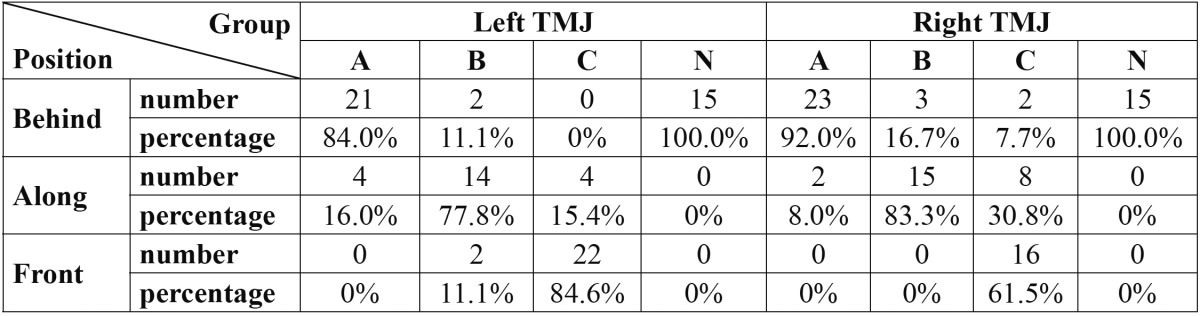
The distribution and proportion of condyle position from articular eminence among all groups.

## Discussion

Few investigations have been dedicated to TMJ hypermobility and its relationship to the position of condyle (1), and most of studies focused on association between GJH and TMDs (2,10-12).

In present study, we compared the anterior, superior and posterior distances of condyle from glenoid fossa in TMJ hypermobile and healthy individuals during MMO.

Winocur E. *et al.* found a positive correlation between hypermobile TMJ and MMO (2). Also, Hircsh C *et al.* concluded that patients with hypermobility had lower risk of having limited mouth opening (13). However, Westling L did not found significant relationship between MMO capacity and peripheral joint mobility (14). The results of present study confirm that there is a relationship between hypermobile TMJ and MMO. Based on the results, TMJ hypermobility was more common in women [74.2%] which is in accordance with some studies (15,16). The superior, posterior and anterior distances were significantly higher in patients with TMJ hypermobility than healthy ones [all *P* values<0.05]. Gateno J *et al.* compared the position of the mandibular condyle in healthy individuals and patients with anterior disc displacement. Their results, which were similar to present results, showed that condyles were positioned more posterior and superior in the fossa in case group than those in control group (17). That might be due to the fact that posterior condylar position is more subjected to physical loadings specially in parafunction activities such as bruxism or excessive mouth opening (18,19). Also, the laxity of ligaments might be the other reason of increasing distance in TMJ and positioning of condyle in front or along to the articular eminence (11). It was observed that the condyle is located mostly behind the articular eminence; as the hypermobility and MMO increased, the condyle was more tended to position along or even in front of articular eminence.

The results of present study revealed that the superior distance was significantly increased as the MMO exceeded, and group C showed significant differences with groups A and B in mentioned distance.

Maybe it is needed to mention that results of some studies reflected that the head of the mandible in hypermobile TMJ was over and sometimes above the lowest point of the eminence articular during MMO (1,20).

Our results were differ from the right TMJ to the left TMJ in some measured distances. It is stated that hypermobility is not an attribute of only the right or left TMJ but it is a characteristic of the masticators as a whole system. Maybe it is better to talk about a hypermobile masticatory system than a single hypermobile TMJ and symptoms of hypermobility might be only obvious in combination with a specific working direction of the masticatory muscles (1).

Although hypermobility is relatively common in the general population, but reports about musculoskeletal complaints are infrequent. As most symptoms are mild and self-limiting so patients may not search for medical attention (21); but it is necessary to note that TMJ hypermobility might result in disk destruction and degenerative disease.

The limitations of the present study were noticed in: uneven sample size, administering one radiograph technique, not observing other movements of mandible due to lack of facilities and etc.

## Conclusions

Considering the mentioned limitations of this study, it can be concluded that TMJ hypermobility showed the characteristic of increased condylar distances in posterior and superior sides between head of condyle and facing wall of glenoid fossa during closed mouth; also, the condyle was more tended to position along or in front of articular eminence specially in higher excessive mouth opening.

## References

[1] Kalaykova SI, Naeije M, Huddleston Slater JJ, Lobbezoo F (2006). Occupational differentiation in dentistry 3. Hypermobility of the temporomandibular joint and condylar position at maximal mouth opening. Ned Tijdschr Tandheelkd.

[2] Winocur E, Gavish A, Halachmi M, Bloom A, Gazit E (2000). Generalized joint laxity and its relation with oral habits and temporomandibular disorders in adolescent girls. J Oral Rehabil.

[3] Pasinato F, Souza JA, Correa EC, Silva AM (2011). Temporomandibular disorder and generalized joint hypermobility: application of diagnostic criteria. Braz J Otorhinolaryngol.

[4] El Hage Y, Politti F, de Sousa DF, Herpich CM, Gloria IP, Gomes CA (2013). Effect of mandibular mobilization on electromyographic signals in muscles of mastication and static balance in individuals with temporomandibular disorder: study protocol for a randomized controlled trial. Trials.

[5] Adair SM, Hecht C (1993). Association of generalized joint hypermobility with history, signs, and symptoms of temporomandibular joint dysfunction in children. Pediatr Dent.

[6] Oral K, Bal Kucuk B, Ebeoglu B, Dincer S (2009). Etiology of temporomandibular disorder pain. Agri.

[7] Hayashi T, Ito J, Koyama J, Hinoki A, Kobayashi F, Torikai Y (1999). Detectability of anterior displacement of the articular disk in the temporomandibular joint on helical computed tomography: the value of open mouth position. Oral Surg Oral Med Oral Pathol Oral Radiol Endod.

[8] Kavuncu V, Sahin S, Kamanli A, Karan A, Aksoy C (2006). The role of systemic hypermobility and condylar hypermobility in temporomandibular joint dysfunction syndrome. Rheumatol Int.

[9] Zawawi KH, Al-Badawi EA, Lobo SL, Melis M, Mehta NR (2003). An index for the measurement of normal maximum mouth opening. J Can Dent Assoc.

[10] Silveira EB, Rocabado M, Russo AK, Cogo JC, Osorio RA (2005). Incidence of systemic joint hypermobility and temporomandibular joint hypermobility in pregnancy. Cranio.

[11] De Coster PJ, Van den Berghe LI, Martens LC (2005). Generalized joint hypermobility and temporomandibular disorders: inherited connective tissue disease as a model with maximum expression. J Orofac Pain.

[12] Kitsoulis P, Marini A, Iliou K, Galani V, Zimpis A, Kanavaros P (2011). Signs and symptoms of temporomandibular joint disorders related to the degree of mouth opening and hearing loss. BMC Ear Nose Throat Disord.

[13] Hirsch C, John MT, Stang A (2008). Association between generalized joint hypermobility and signs and diagnoses of temporomandibular disorders. Eur J Oral Sci.

[14] Westling L, Helkimo E (1992). Maximum jaw opening capacity in adolescents in relation to general joint mobility. J Oral Rehabil.

[15] Michalak M, Paulo M, Bozyk A, Zadrozny L, Wysokinska-Miszczuk J, Michalak I (2013). Incidence of abnormalities in temporomandibular joints in a population of 1,100 urban and rural patients lacking teeth and other parafunctions in 2003-2008. An international problem. Ann Agric Environ Med.

[16] Ozdemir-Karatas M, Peker K, Balik A, Uysal O, Tuncer EB (2013). Identifying potential predictors of pain-related disability in Turkish patients with chronic temporomandibular disorder pain. J Headache Pain.

[17] Gateno J, Anderson PB, Xia JJ, Horng JC, Teichgraeber JF, Liebschner MA (2004). A comparative assessment of mandibular condylar position in patients with anterior disc displacement of the temporomandibular joint. J Oral Maxillofac Surg.

[18] Yang HJ, Kim DS, Yi WJ, Hwang SJ (2013). Reduced joint distance during TMJ movement in the posterior condylar position. J Craniomaxillofac Surg.

[19] Katzberg RW, Tallents RH (2005). Normal and abnormal temporomandibular joint disc and posterior attachment as depicted by magnetic resonance imaging in symptomatic and asymptomatic subjects. J Oral Maxillofac Surg.

[20] Kalaykova S, Naeije M, Huddleston Slater JJ, Lobbezoo F (2006). Is condylar position a predictor for functional signs of TMJ hypermobility?. J Oral Rehabil.

[21] Kirk JA, Ansell BM, Bywaters EG (1967). The hypermobility syndrome. Musculoskeletal complaints associated with generalized joint hypermobility. Ann Rheum Dis.

